# Ferroptosis contributes to hypoxic–ischemic brain injury in neonatal rats: Role of the SIRT1/Nrf2/GPx4 signaling pathway

**DOI:** 10.1111/cns.13973

**Published:** 2022-10-02

**Authors:** Chang Li, Ziyi Wu, Hang Xue, Qiushi Gao, Yahan Zhang, Changming Wang, Ping Zhao

**Affiliations:** ^1^ Department of Anesthesiology Shengjing Hospital of China Medical University Shenyang Liaoning China; ^2^ Department of Anesthesiology People's Hospital of China Medical University (Liaoning Provincial People's Hospital) Shenyang Liaoning China

**Keywords:** ferroptosis, glutathione peroxidase 4, hypoxic–ischemic brain injury, learning and memory impairments, resveratrol

## Abstract

**Aims:**

Hypoxic–ischemic brain injury (HIBI) often results in cognitive impairments. Herein, we investigated the roles of ferroptosis in HIBI and the underlying signaling pathways.

**Methods:**

Ferrostatin‐1 (Fer‐1) or resveratrol (Res) treatments were administered intracerebroventricularly 30 min before HIBI in 7‐day‐old rats. Glutathione peroxidase 4 (GPx4) expression, malondialdehyde (MDA) concentration, iron content, mitochondrial morphology, and the expression of silent information regulator factor 2‐related enzyme 1 (SIRT1) and nuclear factor erythroid‐2‐related factor 2 (Nrf2) were measured after HIBI. Additionally, the weight ratio of left/right hemisphere, brain morphology, Nissl staining, and the Morris water maze test were conducted to estimate brain damage.

**Results:**

At 24‐h post‐HIBI, GPx4 expression was decreased, and MDA concentration and iron content were increased in the hippocampus. HIBI led to mitochondrial atrophy, brain atrophy/damage, and resultant learning and memory impairments, which were alleviated by Fer‐1‐mediated inhibition of ferroptosis. Furthermore, Res‐mediated SIRT1 upregulation increased Nrf2 and GPx4 expression, thereby attenuating ferroptosis, reducing brain atrophy/damage, and improving learning and memory abilities.

**Conclusion:**

The results demonstrated that during HIBI, ferroptosis occurs via the SIRT1/Nrf2/GPx4 signaling pathway, suggesting it as a potential therapeutic target for inhibiting ferroptosis and ameliorating HIBI‐induced cognitive impairments.

## Graphical abstract

Ferroptosis occurs in a neonatal rat model of HIBI. Ferroptosis inhibition via Fer‐1 administration attenuates brain atrophy and learning and memory impairments. Activation of SIRT1/Nrf2/GPx4 signaling following Res treatment represents a potential therapeutic strategy for inhibiting ferroptosis and ameliorating HIBI‐induced learning and memory impairments.

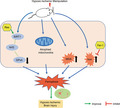

## 1 INTRODUCTION

Hypoxic–ischemic brain injury (HIBI) is mainly caused by perinatal asphyxia and occurs in 2.5/1000 live births, often causing long‐term neurological injury and disorders (e.g., learning and memory impairments and epilepsy).^1^, ^2^, ^3^, ^4^, ^5^ Although therapeutic hypothermia has been clinically approved for HIBI, treatment outcomes are unsatisfactory, with no more than 50% of treated children showing improvement.^6^ Moreover, hypothermic intervention within the optimal treatment window (within 6‐h post‐HIBI) can be difficult, which prevents the intervention from reducing the risk of sudden death in young subjects and leads to further learning impairment.^7^ Furthermore, the development of personalized treatment is curtailed by insufficient knowledge regarding the precise HIBI‐related underlying mechanisms and pathophysiology; therefore, further research is necessary to tailor HIBI‐treatment options.

Ferroptosis, an iron‐dependent form of programmed cell death triggered by glutathione peroxidase 4 (GPx4) inactivation, was first observed in tumor cells in 2012.^8^, ^9^ Characterized by intracellular iron accumulation and lipid peroxidation, ferroptosis is genetically, mechanistically, morphologically, and biochemically distinct from other cell death types.^8^, ^10^, ^11^, ^12^, ^13^ Moreover, ferroptosis does not share any hallmark of other cell death types and can be effectively rescued only through specific inhibitors such as ferrostatin‐1 (Fer‐1).^8^, ^14^, ^15^, ^16^ Although ferroptosis has been identified in several neurological disorders,^17^ its significance in HIBI remains unclear.

Silent information regulator factor 2‐related enzyme 1 (SIRT1), a nicotinamide adenine dinucleotide‐dependent histone deacetylase, regulates cellular responses to injury (e.g., antioxidant defense and apoptosis).^18^, ^19^ It demonstrates neuroprotective roles in various neurodegenerative diseases and ischemic brain injuries. Administration of SIRT1 activators reportedly decreased infarct size and lactate levels, increasing glucose and ATP contents, whereas SIRT1 depletion increased infarct size, as demonstrated in *Sirt1*‐knockout mice.^20^, ^21^, ^22^, ^23^, ^24^ Nuclear factor erythroid‐2‐related factor 2 (Nrf2) drives the antioxidant defense system and can suppress ferroptosis via GPx4 upregulation.^25^ Although SIRT1 enhances Nrf2 expression to exert neuroprotective effects in traumatic brain injury,^26^ the role of SIRT1/Nrf2/GPx4 signal transduction in ferroptosis during HIBI remains unclear.

Here, we assessed ferroptosis based on changes in GPx4 protein expression, iron content, mitochondrial morphology, and malondialdehyde (MDA) concentration in the hippocampus. We hypothesized that ferroptosis occurs in HIBI and contributes to learning and memory impairments. Furthermore, we postulated the involvement of SIRT1/Nrf2/GPx4 signaling and its potential as a therapeutic target.

## 2 MATERIALS AND METHODS

### 2.1 Ethics statement

Adult Sprague–Dawley rats and their pups were housed in specific pathogen‐free animal quarters at 24 ± 1°C with a 12‐h light/dark cycle and ad libitum access to food and water. All animal experiments were performed according to National Institute of Health guidelines and regulations, and the procedures and protocols used in this study were approved by the Animal Ethics Committee of Shengjing Hospital, China Medical University.

### 2.2 Neonatal HIBI model

We prepared a Rice–Vannucci rat model as previously described,^27^, ^28^ with some modifications. Briefly, 7–0 surgical silk was used to permanently ligate the left common carotid artery of 7‐day‐old Sprague–Dawley rats (sex ratio, 1:1) through a small incision in the neck within 5 min after sevoflurane anesthesia. Rats were allowed to recover for 2 h after awakening. Thereafter, the rat pups were placed in a hypoxic chamber under 8% O_2_, 92% N_2_, and 37°C for 2 h.

### 2.3 Drug administration and study group preparation

**FIGURE 1:**
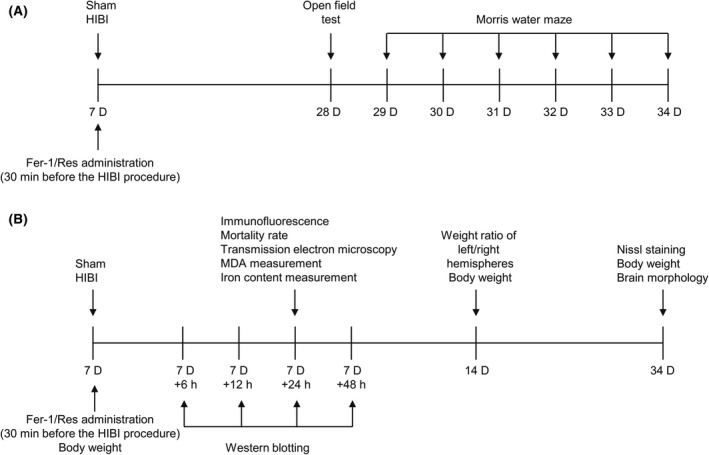
Flowchart of the experiment. HIBI was induced by ischemia and hypoxia manipulation. Rats were randomly intracerebroventricularly injected with Fer‐1 or Res 30 min before HIBI induction. (A) Illustration of behavioral testing. OFT was employed on day 28 post‐birth, and the MWM test was performed on days 29 through 34 post‐birth. (B) Illustration of ferroptosis occurrence, brain damage, and body weight measurement. Western blot was conducted 6, 12, 24, and 48 h after HIBI surgeries to determine when ferroptosis peaked. Rats were sacrificed for a series of experiments (ie immunofluorescence, TEM, MDA assays, and measurements of iron content) at 24‐h post‐HIBI. Body weight measurements were conducted on 7‐, 14‐, and 34‐day post‐birth. The weight ratios of left/right hemisphere measurements were conducted on 14‐day post‐birth. Nissl staining and brain morphology were assessed at 34‐day post‐birth. HIBI, hypoxic–ischemic brain injury; ICV, intracerebroventricular; Fer‐1, ferrostatin‐1; Res, resveratrol; OFT, open‐field test; MWM, Morris water maze; MDA, malondialdehyde; TEM, transmission electron microscopy.

Figure 1 shows a flow chart of the experiment. Ferroptosis was inhibited by administering 3 pmol/3 μl Fer‐1 (HY‐100579; MedChemExpress) to 7‐day‐old Sprague–Dawley rats via intracerebroventricular (ICV) injection 30 min before the HIBI procedure. Fer‐1 was dissolved in saline with 1% dimethyl sulfoxide immediately before injection. Resveratrol (Res; HY‐16561; MedChemExpress) at a concentration of 25 μg/3 μl was dissolved in saline by mechanical agitation for 4 h and administered intracerebroventricularly 30 min before HIBI induction. Experimental rats were randomly divided as follows: (1) Sham group (no treatment), (2) HIBI group (rats exposed to HIBI), (3) HIBI+Fer‐1 group (Fer‐1 injections before HIBI induction), and (4) HIBI+Res group (Res injections before HIBI induction).

### 2.4 Western blotting

The hippocampi of experimental rats were collected 6‐, 12‐, 24‐, and 48‐h post‐HIBI and homogenates were prepared in radioimmunoprecipitation assay lysis buffer (P0013B; Beyotime Biotechnology) with a protease inhibitor cocktail (P8349; Sigma‐Aldrich), followed by determination of protein concentrations using a BCA protein assay kit (P0011; Beyotime Biotechnology). Equal amounts of protein were separated via sodium dodecyl sulfate‐polyacrylamide gel electrophoresis using 12% polyacrylamide gels and then transferred to polyvinylidene fluoride membranes (10,600,023; Cytiva). After blocking with 5% non‐fat dry milk for 2 h, membranes were probed with primary antibodies overnight at 4°C, followed by three washes and incubation with the respective horseradish peroxidase‐conjugated secondary antibodies (Zhongshan Jinqiao Biotechnology Co.) for 2 h at room temperature. Immunoreactivity was visualized using enhanced chemiluminescence, with gray value analysis performed using ImageJ (NIH Image). We used the following primary antibodies for Western blotting: recombinant anti‐GPx4 (1:1000; ab125066; Abcam), anti‐SIRT1 (1:1000; 9475; Cell Signaling Technology), anti‐Nrf2 (1:1000; 16396‐1‐AP; Proteintech), and anti‐glyceraldehyde 3‐phosphate dehydrogenase (1:1000; 60004‐1‐Ig; Proteintech).

### 2.5 Immunofluorescence

Rat brains were harvested following transcardial perfusion with normal saline and 4% paraformaldehyde under deep anesthesia 24‐h post‐HIBI. Specimens were fixed in 4% paraformaldehyde for 24–48 h, dehydrated in a graded ethanol series, and paraffin‐embedded. Coronal sections (3.5 μm thick) were then prepared. After deparaffinization and antigen retrieval, sections were probed with 10% fetal bovine serum in phosphate‐buffered saline (PBS) for 40 min and incubated with primary anti‐GPx4 (1:200; ab125066; Abcam) and anti‐NeuN (1:200; ab104224; Abcam) antibodies overnight at 4°C. Thereafter, sections were washed three times and incubated for 4 h at room temperature with tetramethyl rhodamine‐isothiocyanate‐conjugated goat anti‐mouse IgG (1:200; SA00007‐1; Proteintech) and fluorescein isothiocyanate‐conjugated goat anti‐rabbit IgG (1:200; A22120‐1; Abbkine, Wuhan, China) secondary antibodies. Nuclei were then stained with 4′,6‐diamidino‐2‐phenylindole (C1006; Beyotime Biotechnology) for 5 min, and immunofluorescence images were captured using a Nikon C1Si confocal microscope.

### 2.6 Determination of body weight, mortality rate, and weight ratio of left/right hemisphere

Mortality rate was calculated by recording the death of rats during the period from HIBI to 34‐day post‐birth. We measured the bodyweight of rats from all groups on days 7, 14, and 34 after birth. Cerebral hemispheres were dissected and weighed 7‐day post‐HIBI for calculation of the weight ratio of left/right hemisphere.

### 2.7 Transmission electron microscopy (TEM)

Hippocampi from harvested brains were carefully dissected and fixed in TEM fixative (G1102; Servicebio), followed by fixation in 1% osmium tetroxide. Thereafter, sections (60–80 nm thick) were stained with uranyl acetate and lead citrate after dehydration, infiltration, and embedding. TEM images were captured using a HT7700 microscope.

### 2.8 MDA measurement

To evaluate lipid peroxidation in hippocampal samples, we used an MDA assay kit (S0131; Beyotime Biotechnology) to measure MDA concentration. Briefly, each hippocampus was weighed before preparing a homogenate (containing 10% hippocampal‐tissue weight) with PBS on ice, which was centrifuged (12,000 ***g***, 10 min, 4°C) to obtain the supernatant. Following the addition of assay reagents, the mixtures were heated (100°C, 15 min), and the supernatant was obtained by centrifugation (1000 ***g***, 10 min) after cooling to room temperature. MDA concentration was determined by measuring absorbance at 532 nm.

### 2.9 Inductively coupled plasma mass spectrometry (ICP‐MS)

Total iron content in the hippocampus was measured using ICP‐MS. Hippocampi were weighed before transfer to microwave digestion tubes containing 5 ml nitric acid and 2 ml of 30% H_2_O_2_, followed by digestion in a microwave system for 1.5 h (blank control: 5 ml nitric acid and 2 ml of 30% H_2_O_2_). Once the temperature of the acid‐driving instrument reached 160°C, prepared tubes were inserted and heated until the solutions were reduced to <0.5 ml. The residual samples were diluted to 30 ml with ultra‐pure water and assessed using a 7700x ICP‐MS system (Agilent Technologies).

### 2.10 Open‐field test (OFT)

To test exploratory behavior and spontaneous activity, an OFT was conducted using a black square open‐field apparatus (100 × 100 × 45 cm) on day 28 post‐birth. The floor was marked into central and border zones and cleaned with 70% ethanol between tests. Rats were subjected to the OFT and their activity was monitored for 10 min using the EthoVision XT video tracking system (Noldus), with movement, distance traveled, and average speed recorded.

### 2.11 Morris water maze (MWM)

To evaluate spatial learning ability and memory following HIBI, MWM tests were performed on days 29 through 34 post‐birth. A circular pool (diameter, 160 cm; height, 60 cm) with black walls was used, with water poured into the pool to a depth 1.5 cm higher than a removable escape platform (diameter, 12 cm). The whole experiment was performed in warm water (20 ± 1°C). Training sessions were conducted four times per day for 5 days. Eight rats were individually placed into the water from different quadrants and permitted to search for the platform for up to 90 s. Rats failing to locate the platform were guided to it. All rats were coaxed to remain on the platform for 20 s. Escape latency was defined as the time required to reach the platform. For rats unable to find the platform within the allotted time, escape latency was recorded as 90 s. On day 34 post‐birth, rats were released into the water and allowed to swim freely for 90 s in the absence of the platform. Escape latency, swim paths, swimming speed, and other relevant parameters were monitored and analyzed using an automated video tracking system (Shanghai Mobile Datum Ltd.).

### 2.12 Nissl staining

After the behavioral tests, brains were harvested according to the procedure described for the immunofluorescence assay. Nissl staining was conducted on 3.5‐μm‐thick coronal sections taken from approximately 3.3 mm caudal to the bregma. Representative images were captured under a Nikon Eclipse C1 microscope. Neuronal density in the cornu ammonis 1 (CA1) region was calculated by counting the number of Nissl‐positive neurons.

### 2.13 Statistical analyses

Data represent the mean ± the standard deviation (SD). Statistical analysis was performed using SPSS (v17.0; SPSS Inc.). We used the Shapiro–Wilk test to assess the normality of the distribution of continuous variables, and Student's *t* test (two‐tailed) and one‐way analysis of variance (anova), followed by Tukey's post‐hoc test, were used to compare the means of two and multiple groups, respectively, for normally distributed data. Alternatively, Mann–Whitney *U* test and Kruskal–Wallis *H* test were used to compare data of two and multiple groups, respectively. Escape latency determined during the MWM tests was analyzed using two‐way repeated‐measures anova. A *Z* test was used to analyze mortality among multiple groups. A *p* < 0.05 was considered significant.

## 3 RESULTS

Table 1 and Table S1 show the general characteristics of different study groups. Mortality after HIBI in each group was approximately 10% and not significantly altered by administering different drugs. We observed no significant differences in body weight between time points (7‐, 14‐, and 34‐day post‐birth).

**TABLE 1:** General characteristics (mean ± SD)

	Fate after HIBI			Body weight (g) of the survivors		
	Total	Dead	Mortality (%)	7 days old	14 days old	34 days old
Sham	40	0	0	13.4 ± 0.8	32.4 ± 1.8	92.5 ± 2.3
HIBI	40	4	10	13.7 ± 0.0.6	31.5 ± 1.3	90.3 ± 1.7
HIBI+Fer‐1	40	3	7.5	14.1 ± 1.3	30.4 ± 1.3	91.1 ± 1.5
HIBI+ Res	40	2	5	14.0 ± 0.7	31.6 ± 1.5	91.3 ± 4.8

Abbreviations: HIBI, hypoxic–ischemic brain injury; Fer‐1, ferrostatin‐1; Res, resveratrol.

### 3.1 Ferroptosis is involved in HIBI, reaching peak levels in the hippocampus at 24‐h post‐event

**FIGURE 2:**
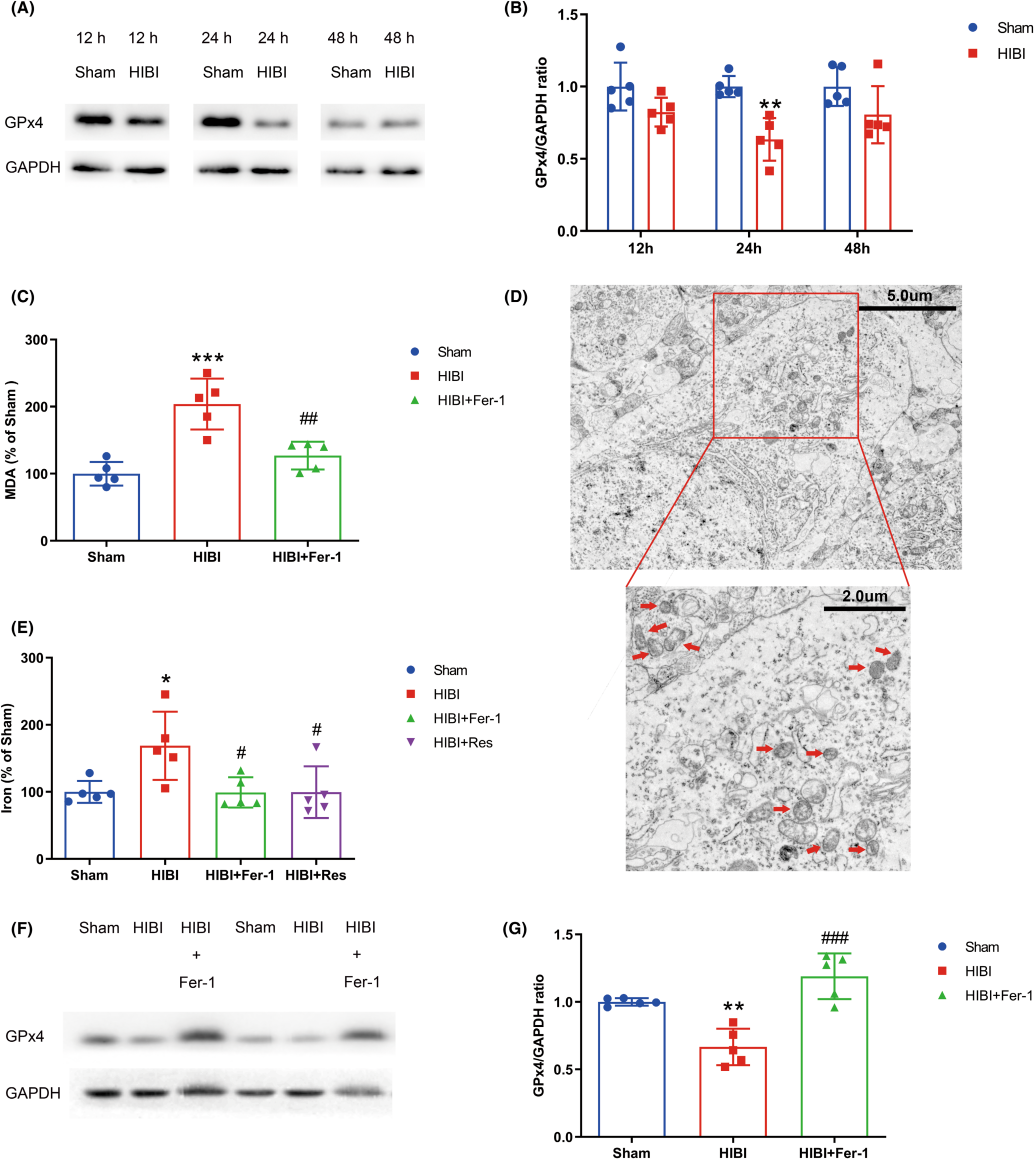
Ferroptosis occurred in HIBI and peaked at 24‐h post‐event. (A) Western blot results indicated a rapid decline in GPx4 levels in rat hippocampi at 24‐h post‐HIBI (*n* = 5 for each time point). (B) Quantification of GPx4 level. (C) Lipid peroxidation assay results revealed an increase in MDA concentration at 24‐h post‐HIBI and a decrease following Fer‐1 administration relative to the Sham and HIBI groups, respectively (*n* = 5 per group). (D) Representative TEM images of hippocampal neuron mitochondria at 24‐h post‐HIBI. The atrophied mitochondria are indicated by red arrows (*n* = 3 per group). (E) ICP‐MS results revealed increases in iron content at 24‐h post‐HIBI and declines in iron content following Fer‐1 or Res administration (*n* = 5 per group). (F) Western blot results indicated alleviation of GPx4 downregulation in the HIBI group following Fer‐1 administration (*n* = 5 per group). (G) Quantification of GPx4 levels. Values are presented as the mean ± SD. *p < 0.05, **p < 0.01, ***p < 0.001 vs. the Sham group; #*p* < 0.05, ##*p* < 0.01, and ###*p* < 0.001 vs. the HIBI group. HIBI, hypoxic–ischemic brain injury; GPx4, glutathione peroxidase 4; Fer‐1, ferrostatin‐1; Res, resveratrol; MDA, malondialdehyde; TEM, transmission electron microscopy; ICP‐MS, inductively coupled plasma mass spectrometry.

To confirm ferroptosis occurrence and peak time in HIBI, we assessed time‐course expression of GPx4 using Western blot. We found a significant decrease in GPx4 levels 24‐h post‐HIBI (Figure 2A,B), indicating ferroptosis peaked within the hippocampus at 24‐h post‐event. GPx4 is widely used as a ferroptosis marker due to the strong inverse association of its protein levels with ferroptosis.^29^, ^30^ Based on these findings, we performed subsequent experiments using hippocampi collected 24‐h post‐HIBI.

**FIGURE 3:**
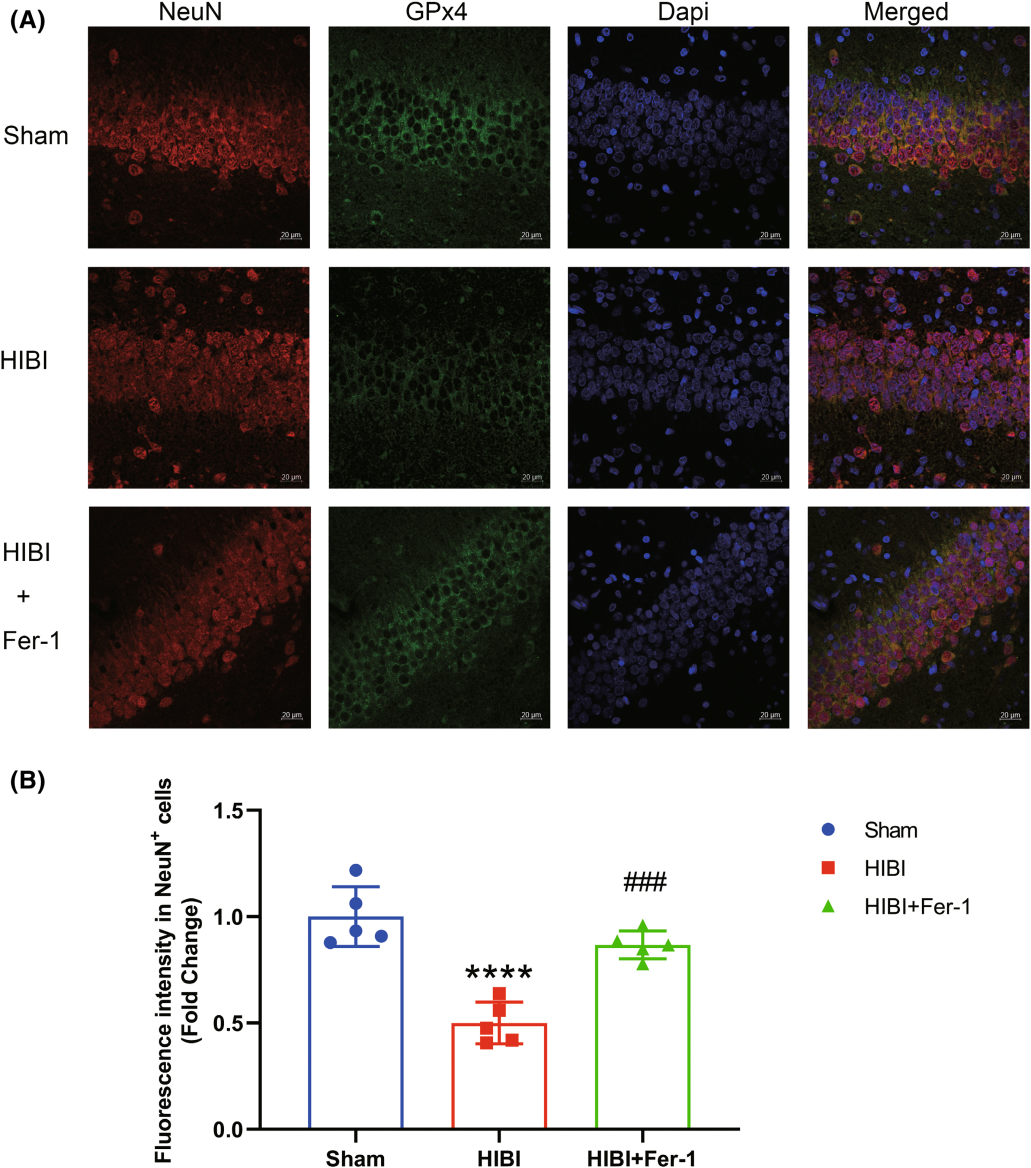
Ferroptosis occurred in hippocampal CA1 region neurons after HIBI. (A) Representative immunofluorescence staining of GPx4 (green) and NeuN (red) in the hippocampal CA1 region. Double labeling confirmed downregulation of GPx4 expression in NeuN‐positive neurons within the hippocampal CA1 region in the HIBI group, whereas Fer‐1 administration upregulated GPx4 expression in neurons within the hippocampal CA1 region (*n* = 5 per group). Scale bar = 20 μm. (B) Fluorescence intensity of GPx4 level in NeuN‐positive cells was quantified in the Sham, HIBI, and HIBI+Fer‐1 groups. Values are presented as the mean ± SD. ****p < 0.0001 vs. the Sham group; ###*p* < 0.001 vs. the HIBI group. HIBI, hypoxic–ischemic brain injury; GPx4, glutathione peroxidase 4; Fer‐1, ferrostatin‐1; ICV, intracerebroventricular; CA1, cornu ammonis 1.

We examined the iron content and lipid‐peroxidation levels in the hippocampus using ICP‐MS and MDA assays, respectively. Both MDA concentration and iron content in the hippocampus were significantly higher in HIBI‐exposed groups relative to the Sham group (Figure 2C,E). Furthermore, TEM observation of mitochondrial morphology in hippocampal nerve cells in the HIBI group identified atrophied mitochondria with increased membrane density, fewer cristae, and ruptured outer membranes (Figure 2D). Additionally, immunofluorescence revealed GPx4 downregulation in neurons within the hippocampal CA1 region in the HIBI group, indicating that CA1 neurons underwent ferroptosis following HIBI (Figure 3A,B).

We administered the ferroptosis inhibitor Fer‐1 via ICV injection 30 min before HIBI to confirm ferroptosis occurrence during HIBI. Fer‐1 administration attenuated HIBI‐induced iron and MDA accumulations and GPx4 downregulation (Figure 2C,E–G), with immunofluorescence confirming Fer‐1‐mediated rescue of GPx4 downregulation in neurons (Figure 3A,B). Notably, we observed no significant differences in iron content, MDA concentration or GPx4 expression between the Sham group and those receiving Fer‐1 ICV injection (Figure S1A–D). Moreover, Fer‐1 treatment did not significantly change GPx4 expression levels in neurons in Sham group rats (Figure S1E,F). These results indicated that ferroptosis occurred during HIBI and peaked 24‐h post‐event.

### 3.2 Ferroptosis contributes to HIBI‐induced brain atrophy/damage

**FIGURE 4:**
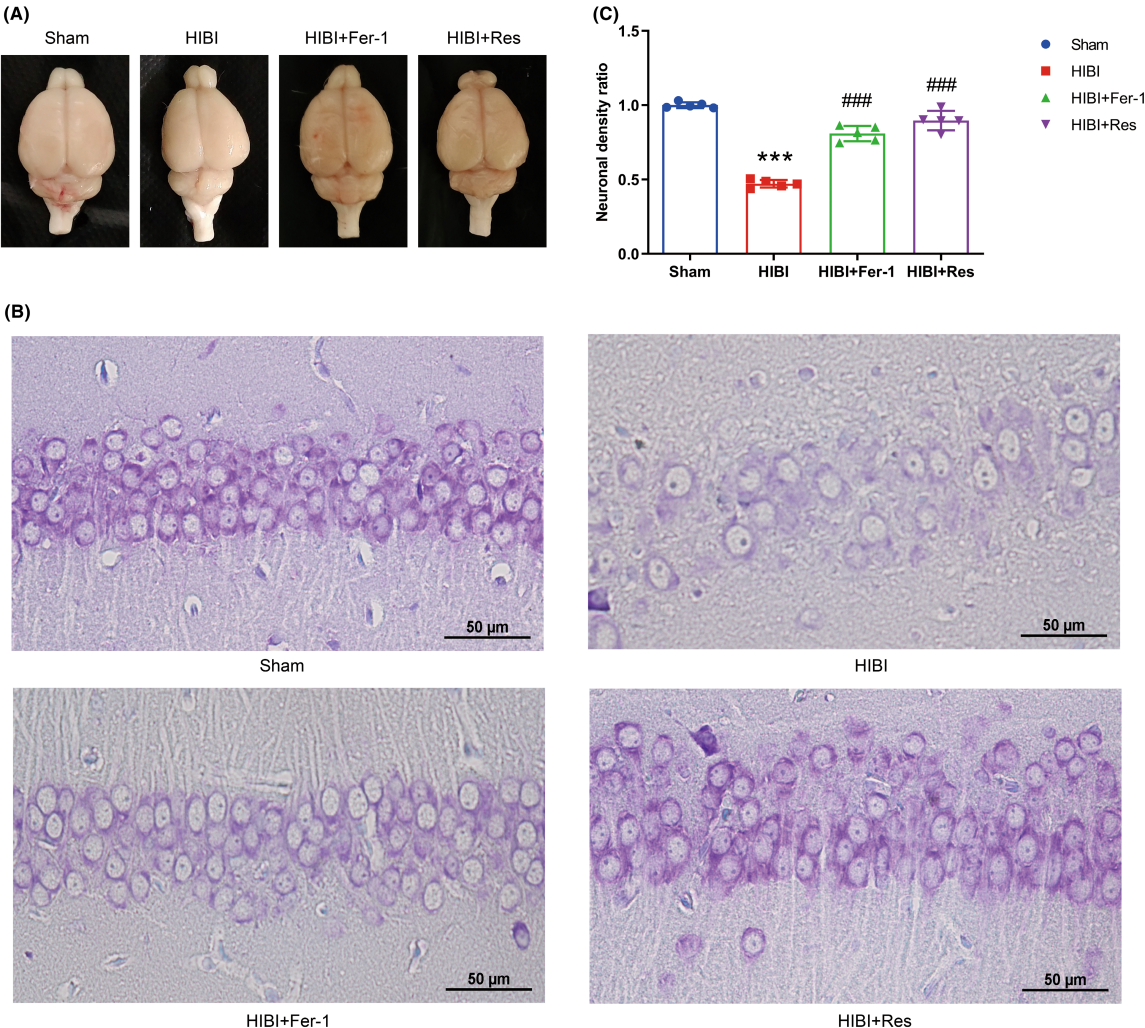
Fer‐1 or Res administration protected against HIBI‐induced brain atrophy/damage. (A) Representative brain morphological images taken on day 34 post‐birth. Fer‐1 or Res administration protects against HIBI‐induced loss of brain matter. (B) Nissl staining in the hippocampal CA1 region on day 34 post‐birth revealed that HIBI resulted in irregular neuronal arrangement and an increase in inter‐neuronal space, both of which were alleviated following Fer‐1 or Res administration (*n* = 5 per group). Scale bar = 50 μm. (C) The neuronal density ratio in the hippocampal CA1 region indicated lower neuronal density in the HIBI group, which was improved after Fer‐1 or Res treatment (*n* = 5 per group). Data represent the mean ± SD. ***p < 0.001 vs. the Sham group; ###*p* < 0.001 vs. the HIBI group. HIBI, hypoxic–ischemic brain injury; GPx4, glutathione peroxidase 4; Fer‐1, ferrostatin‐1; Res, resveratrol; CA1, cornu ammonis 1.

We examined HIBI‐induced brain atrophy/damage at 28‐day post‐HIBI. Figure 4A shows representative images. Compared with the Sham group, the HIBI group presented atrophied left hemispheres, which was attenuated by Fer‐1 administration, with similarly altered brain morphology observed between the Sham and Sham+Fer‐1 groups (Figure S2A). Given the efficacy of the weight ratio of left/right hemisphere as an index of atrophy,^31^ we calculated these ratios at 7‐day post‐HIBI. The results revealed a significant loss of brain tissue on the ischemic side in the HIBI group, which was prevented by Fer‐1 treatment in the HIBI+Fer‐1 group (Table 2). Notably, we observed no significant differences in weight ratios of left/right hemisphere between the Sham group and those receiving Fer‐1 ICV injection (Table S2).

**TABLE 2:** Weight ratio of left/right cerebral hemispheres (mean ± SD)

	Left cerebral weight (mg)	Right cerebral weight (mg)	Left/right (%)
Sham	479.4 ± 27.63	464.8 ± 25.70	103.1 ± 1.75
HIBI	365.8 ± 33.19**	417.6 ± 38.86	87.62 ± 0.86***
HIBI+ Fer‐1	484.4 ± 40.04^##^	485.8 ± 27.25	99.59 ± 2.87^###^
HIBI+ Res	467.0 ± 63.03^##^	469.8 ± 52.85	99.23 ± 2.76^###^

** *p* < 0.01.

*** *p* < 0.001 vs. the Sham group.

^##^ *p* < 0.010.

^###^ *p* < 0.001 vs. the HIBI group.

We employed Nissl staining to evaluate hippocampal architecture. Healthy Nissl‐stained cells were characterized by the presence of Nissl substance, abundant cytoplasm, and oval nuclei. Digital images revealed disordered neurons in the CA1 region of HIBI group hippocampi, with increased space between neurons and decreased cell density relative to the Sham group (Figure 4B,C). Conversely, CA1 neurons from HIBI+Fer‐1 group rats exhibited a more ordered distribution, decreased space between neurons, and increased cell density relative to the HIBI group (Figure 4B,C). Additionally, we observed no significant difference in cell density or hippocampal architecture of the CA1 region between the Sham and Sham +Fer‐1 groups (Figure S2B,C).

### 3.3 Ferroptosis contributes to HIBI‐related learning and memory impairments

**FIGURE 5:**
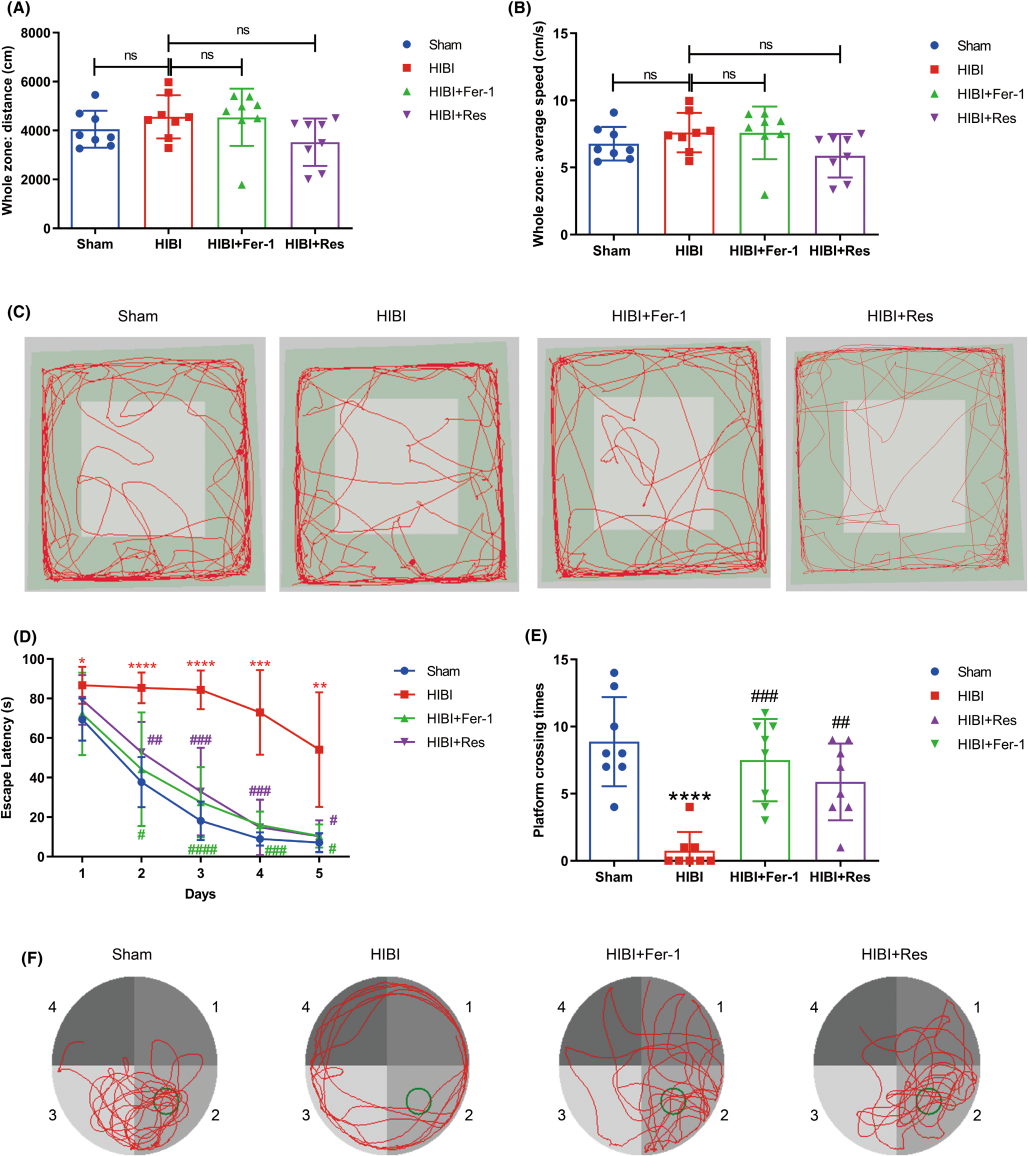
MWM tests and OFTs in the Sham, HIBI, HIBI+Fer‐1, and HIBI + Res groups. (A,B) Statistical results of total distance and average speed measured during the OFT (*n* = 8 per group). (C) Representative behavioral tracks of rats in different groups during the OFT. (D) Measurements of escape latency indicated that rats in the HIBI group exhibited poor spatial data acquisition ability, which was improved by Fer‐1 or Res administration in the HIBI+Fer‐1 and HIBI+Res groups, respectively (*n* = 8 per group). (E) Platform crossing times indicated that rats in the HIBI+Fer‐1 and HIBI+Res groups exhibited better memory retention than those in the HIBI group (*n* = 8 per group). (F) Representative swimming tracks of Sham, HIBI, HIBI+Fer‐1, and HIBI+Res rats. Data represent the mean ± SD. ns: not significant. *p < 0.05, **p < 0.01, ***p < 0.001, ****p < 0.0001 vs. the Sham group; #*p* < 0.05, ##*p* < 0.01 ###*p* < 0.001, ####*p* < 0.0001 vs. the HIBI group. HIBI, hypoxic–ischemic brain injury; Fer‐1, ferrostatin‐1; Res, resveratrol; OFT, open‐field test; MWM, Morris water maze; CA1, cornu ammonis 1.

OFT results indicated non‐significant differences in motor function among the Sham, HIBI, and HIBI+Fer‐1 groups based on total distance and average speed recordings (Figure 5A–C). MWM test results indicated that Fer‐1 administration reduced the prolonged escape latency relative to the HIBI group (Figure 5D). Following platform removal on day 6 of the MWM tests, the platform crossing times of the HIBI group decreased relative to those of the Sham group but were increased by ICV administration of Fer‐1 (HIBI+Fer‐1 group) (Figure 5E). Moreover, the swimming tracks recorded for HIBI group rats were more disordered than those of their Sham counterparts, whereas those of HIBI+Fer‐1 rats were more orderly (Figure 5F). Additionally, results of the OFT and MWM tests indicated non‐significant differences between the Sham and Sham +Fer‐1 groups, suggesting that Fer‐1 treatment did not significantly change long‐term learning or memory ability in Sham group rats (Figure S3A–D). These findings suggested that HIBI‐induced ferroptosis resulted in long‐term learning and memory impairments, which could be ameliorated by Fer‐1‐mediated inhibition of ferroptosis.

### 3.4 SIRT1/Nrf2/GPx4 signaling is involved in ferroptosis during HIBI

**FIGURE 6:**
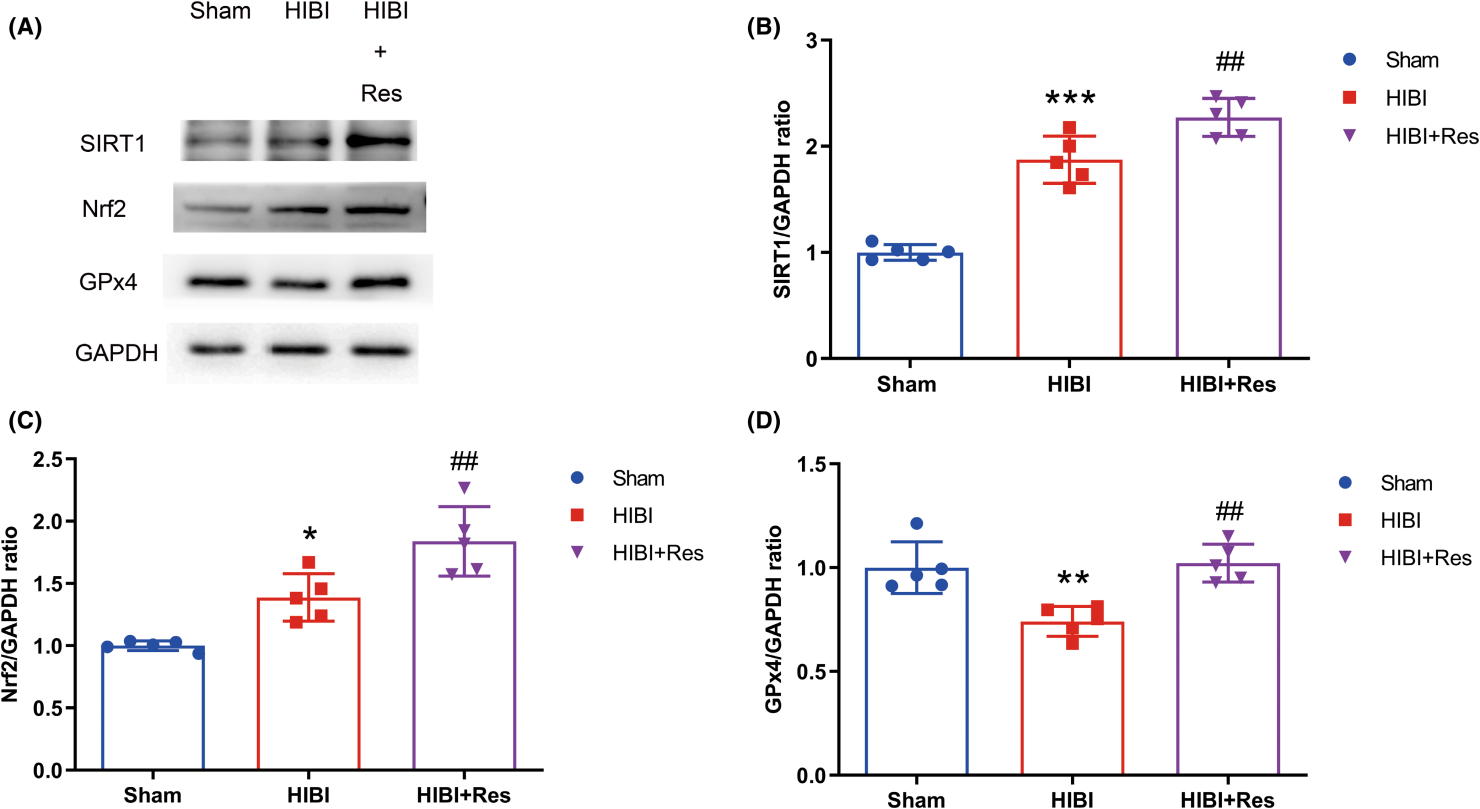
Role of the SIRT1/Nrf2/GPx4 signaling pathway in ferroptosis. (A) Western blot analysis of SIRT1, Nrf2, and GPx4 expression indicated upregulation of SIRT1 and Nrf2 and downregulation of GPx4 after HIBI induction. SIRT1, Nrf2, and GPx4 expression was upregulated after Res administration (*n* = 5 per group). (B) Quantification of SIRT1. (C,D) Quantification of (C) Nrf2 and (D) GPx4. Data represent the mean ± SD. *p < 0.05, **p < 0.01, ***p < 0.001 vs. the Sham group; ##*p* < 0.01 vs. the HIBI group. HIBI, hypoxic–ischemic brain injury; GPx4, glutathione peroxidase 4; Fer‐1, ferrostatin‐1; SIRT1, silent information regulator factor 2‐related enzyme 1; Nrf2, nuclear factor erythroid‐2‐related factor 2; Res, resveratrol; GAPDH, glyceraldehyde 3‐phosphate dehydrogenase; CA1, cornu ammonis 1.

To investigate the involvement of SIRT1/Nrf2/GPx4 signaling in HIBI‐induced ferroptosis, we administered the SIRT1 agonist Res to rats in the HIBI+Res group and evaluated SIRT1, Nrf2, and GPx4 levels in Sham, HIBI, and HIBI+Res rats hippocampi via Western blot. HIBI stimulated SIRT1 and Nrf2 expression and significantly reduced GPx4 expression relative to the Sham group, whereas Res administration markedly increased SIRT1, Nrf2, and GPx4 expression relative to the HIBI group (Figure 6A–D). Additionally, we found no significant difference in SIRT1, Nrf2, or GPx4 levels between the Sham and Sham+Res groups (Figure S4A–D). Furthermore, the results revealed lower iron content in the HIBI+Res group than in the HIBI group (Figure 2E), and Res treatment did not significantly change the iron content in Sham group rats (Figure S1A). These results indicated that Res treatment suppressed ferroptosis via the SIRT1/Nrf2/GPx4 signaling pathway.

### 3.5 Res, a SIRT1 agonist, limits the degree of HIBI‐related brain atrophy/damage

Figure 4A shows comparative images of cerebral morphology among different groups, indicating the degree of brain damage. ICV injection of Res limited the degree of atrophy in the left hemisphere relative to that in the HIBI group but did not alter brain morphology in the Sham group (Figure S2A). Similarly, weight ratios of left/right hemisphere increased significantly after Res treatment as compared with those in the HIBI group, confirming that Res treatment alleviated brain atrophy/damage (Table 2). However, Res treatment did not alter weight ratios of left/right hemisphere in the Sham group (Table S2).

Nissl staining revealed more orderly arrangements of CA1 regional neurons of the HIBI+Res group with less inter‐neuronal space than those of the HIBI group (Figure 4B). Moreover, CA1 regional neurons of the HIBI+Res group had greater cell density than those of the HIBI group (Figure 4C). Furthermore, ICV injection of Res did not significantly change cell density or hippocampal architecture of the CA1 region in Sham group rats (Figure S2B,C).

### 3.6 Res treatment alleviates HIBI‐related learning and memory impairments

We conducted behavioral tests to determine the involvement of SIRT1/Nrf2/GPx4 signaling in HIBI‐induced learning and memory impairments and assess its relationship to ferroptosis. Based on average speed and total distance, OFT results (Figure 5A–C) revealed no differences in motor function between groups. Moreover, MWM results indicated significant decreases in average escape latencies (Figure 5D), as well as more ordered swimming tracks, in the HIBI+Res group relative to the HIBI group (Figure 5F). Furthermore, Res treatment increased platform crossing frequency (Figure 5E). Notably, we found non‐significant differences in OFT and MWM test results between the Sham and Sham+Res groups (Figure S3A–D).

## 4 DISCUSSION

HIBI is a common form of neonatal brain injury that poses a high risk of lifelong neurological impairment.^3^, ^32^ The principal findings of the present study are as follows: (i) ferroptosis peaked at 24‐h post‐HIBI and contributed to brain damage, as well as learning and memory impairments; (ii) Fer‐1 administration suppressed ferroptosis and protected the brain from HIBI to a certain extent; (iii) SIRT1/Nrf2/GPx4 signaling was involved in HIBI‐induced ferroptosis; and (iv) Res exerted a neuroprotective effect by inhibiting neuronal ferroptosis, highlighting the latter as a potential therapeutic target for HIBI treatment.

Ferroptosis can be triggered by inhibiting system X_C_^−^, GPx4 inactivation, reactive oxygen species and iron accumulation, and excessive lipid peroxidation.^33^, ^34^, ^35^, ^36^, ^37^, ^38^ Additionally, ferroptosis occurs in various neurological disorders with various underlying mechanisms.^17^, ^39^ Traumatic brain injury can result in lipid‐ROS and iron accumulation, dysfunctional iron metabolism, reduced activity of glutathione peroxidase (GPx), and, ultimately, ferroptosis.^40^ Qu et al^41^ found that ferroptosis was induced after subarachnoid hemorrhage by the elevated expression of Acyl‐CoA synthetase long‐chain family member 4 (ACSL4); inhibiting its expression or suppressing ferroptosis attenuated tissue damage and improved long‐term outcomes. A recent study showed that selenium treatment promoted GPx4 expression and inhibited ferroptosis in hemorrhagic stroke models.^42^ Moreover, intraperitoneal injection of a Tat‐linked selenoprotein P Peptide increased GPx4 expression, thus resisting ferroptosis in a rodent model of focal ischemia.^42^ Finally, Ge MH et al. found that zinc inhibited spinal cord injury‐induced ferroptosis by increasing Nrf2 and HO‐1 expression and activating the GPx4 signaling pathway^43^; Administering Brusatol (an Nrf2 inhibitor) significantly reversed the effects of zinc.^43^

Although previous studies have explored the relationship between ferroptosis and HIBI, the mechanisms underlying HIBI occurrence and development remain unclear, thereby compromising treatment options. The neonatal brain is characterized by increased oxygen consumption, high levels of unsaturated fatty acids, and low antioxidant levels, making it more vulnerable to oxidative stress.^44^ Because ferroptosis occurs when the cellular antioxidant defense system is overwhelmed,^17^ we inferred that ferroptosis might take place in the brains of newborns that have suffered HIBI. Moreover, previous reports have suggested that ischemia can result in iron accumulation within the acidulated environment, which, together with glutamate accumulation after HIBI, can inhibit system X_C_^−^ and then induce ferroptosis.^38^, ^45^ Ferroptosis may be closely related to brain development, and genes associated with ferroptosis are highly expressed in the embryonic and postnatal brain.^46^ Embryonically, the expression of ferroptosis‐related genes such as SLC7A11 and HMOX1 is increased, promoting nerve‐cell proliferation and cognitive development.^47^ In addition, iron is an essential nutrient for neurodevelopment in infants; however, excess iron accumulation is harmful to brain development.^48^ Postnatally, the expression of transferrin and its receptors is high in the brain^47^ and iron moves out of the blood by binding to transferrin and into the brain by binding to transferrin receptors on microvascular endothelial cells, thus maintaining iron homeostasis.^49^ After brain ischemia, excess iron accumulates in the acidic environment, which negatively affects brain development and leads to ferroptosis.^38^, ^45^ Moreover, the expression levels of ferroptosis‐related genes (SLC7A11 and GPx4) were decreased, thus leading to ferroptosis and a decline in cognitive function.^50^ In addition, inhibiting ferroptosis improved the neurodevelopment and cognitive abilities of HIBI.^33^ Altogether, these findings illustrate that ferroptosis is closely related to brain development.

On the other hand, once aberrant neuronal cell death such as ferroptosis occurs, the injured neurons cannot be replenished due to the lack of splitting ability and, therefore, brain development is negatively affected.^51^ Inhibition of ferroptosis and necroptosis each reportedly reduce neuronal death by more than 80%, reflecting the pivotal role of ferroptosis in brain injury.^34^ Studies have illustrated that various forms of cell death occur successively during brain injury, explaining the increased neuroprotection of combined application of several cell death inhibitors relative to a single type.^8^, ^14^, ^15^, ^52^, ^53^, ^54^, ^55^ Further investigation into the exact timing of different cell death types and their interaction is crucial for enabling more precise and effective medical intervention. Ferroptosis may represent a potential therapeutic target for cerebral ischemia treatment.^33^, ^56^, ^57^

The present results provide evidence of the involvement of ferroptosis in HIBI, with peak levels observed 24‐h post‐injury, suggesting that ferroptosis inhibition protects the brain against HIBI‐induced damage. GPx4 downregulation can serve as a key marker of ferroptosis.^58^ Inhibition of GPx4 activity induces lipid peroxidation and cell death, and its deletion is embryonically lethal.^9^, ^59^ Additionally, the sudden death of CA1 hippocampal neurons occurs following conditional *Gpx4* knockout, highlighting the heightened sensitivity of neurons to GPx4 deletion.^60^ In the present study, we observed significant declines in GPx4 expression within the hippocampal CA1 region, which is closely associated with learning and memory. These results agree with previous reports on ferroptosis occurrence in response to significantly lower GPx4 expression in ischemic rat models.^61^ However, another study reported increased GPx4 expression 6 h after intracerebral hemorrhage (ICH) induction.^42^ These conflicting results might be explained by ICH‐induced increases in GPx4 expression representing an insufficient adaptive homeostatic response to withstanding ferroptosis. Consistent with this possibility, we demonstrated transient increases in GPx4 levels at 6‐h post‐HIBI, although the change was not significant (Figure S5). Furthermore, we identified elevated MDA concentration and iron content at 24‐h post‐HIBI. Moreover, we observed typical morphological characteristics of ferroptosis, including mitochondrial shrinkage, loss of mitochondrial cristae, and rupture of mitochondrial outer membranes, in hippocampi collected 24‐h post‐HIBI, supporting the involvement of ferroptosis in HIBI‐related pathophysiology. Importantly, differences in left/right hemisphere weight ratios and neuronal density indicated severe brain damage in rats from the HIBI group, as well as poor long‐term learning and memory abilities. Notably, Fer‐1 administration increased GPx4 levels, reduced MDA concentration and iron content, enhanced GPx4 expression in neurons, and attenuated brain loss/damage and learning and memory impairments. These results identified the involvement of neuronal ferroptosis in HIBI and its contribution to HIBI‐induced learning and memory impairments.

Nrf2, a master transcriptional regulator of antioxidation, can be stimulated by accumulating ROS during HIBI and, subsequently, induces the expression of antioxidative genes to reduce ROS levels.^62^, ^63^ Nrf2 regulates the expression of ferroptosis‐related factors, and ferroptosis can be inhibited through Nrf2 upregulation.^64^, ^65^, ^66^ SIRT1 is closely associated with cell senescence and oxidative stress and can protect the brain from hypoxic–ischemic injury.^18^, ^19^, ^24^, ^26^ Furthermore, mounting evidence indicates that SIRT1 can enhance the expression and transcriptional activity of Nrf2 to exert potent antioxidant effects.^67^ In the present study, SIRT1 and Nrf2 expression was stimulated in response to HIBI because of the adaptive homeostatic response to ROS; however, their elevated expression was insufficient to resist HIBI, which caused a decline in GPx4 (a ferroptosis marker) expression and subsequent ferroptosis. Notably, Res‐mediated SIRT1 activation was stronger than HIBI alone and, as expected, resulted in a higher Nrf2 and GPx4 expression, ameliorating HIBI‐induced ferroptosis and exerting a neuroprotective effect. These observations propose Res as a promising therapeutic agent for HIBI that could alleviate HIBI‐related effects by inhibiting ferroptosis via the SIRT1/Nrf2/GPx4 signaling pathway.

This study has limitations. We did not assess the acute phase of brain injury after HIBI but rather monitored injury on days 7 and 28 post‐HIBI using the weight ratios of left/right hemisphere, MWM tests, Nissl staining, and morphological assessments. Second, we administered Fer‐1 and Res intracerebroventricularly, which is not extensively employed in clinical practice. Third, although we focused on SIRT1/Nrf2/GPx4 signaling, other pathways may be related to ferroptosis. Finally, as the brain develops towards adulthood, sex influences cerebral ischemia outcomes.^68^ Female mice showed less brain damage than male mice following global cerebral ischemia, and the incidence of vascular events was lower in female stroke‐prone hypertensive rats than that in males after focal cerebral ischemia.^69^, ^70^ Sex dimorphism also exists in dopamine neurotransmission, resulting in males being more sensitive to reward and females to punishment.^71^ Moreover, sex influences both brain microvessels and functional improvement after cerebral ischemia.^72^, ^73^ However, our experiments were carried out in both male and female rats at an equal ratio of 1:1, and we did not explore the impact of sex differences in HIBI‐related long‐term learning and memory abilities. Future studies should address these limitations to validate the present findings.

## 5 CONCLUSION

The present study demonstrated that ferroptosis occurred in a neonatal rat model of HIBI, reaching peak levels at 24‐h post‐induction. Ferroptosis inhibition via Fer‐1 attenuated brain atrophy and learning and memory impairments. The SIRT1/Nrf2/GPx4 signaling pathway was implicated in ferroptosis, and its activation via Res administration exerted neuroprotective effects. These results highlight strategies for the clinical treatment of HIBI, particularly via ferroptosis inhibition.

## CONFLICT OF INTEREST

The authors declare no conflict of interests.

## Supporting information


Figure S1

Figure S2

Figure S3

Figure S4

Figure S5

Figure S6

Tables S1‐S2

## Data Availability

The data that support the findings of this study are available from the corresponding author upon reasonable request.
