# Supplementary material for: Ferroptosis contributes to hypoxic–ischemic brain injury in neonatal rats: Role of the SIRT1/Nrf2/GPx4 signaling pathway
Source: CNS Neurosci Ther. 2022 Oct 2;28(12):2268–80. doi: 10.1111/cns.13973 (PMC9627393; doi:10.1111/cns.13973)
Supplement: Supplementary file 1 — Figure S1 [file CNS-28-2268-s002.doc]

**
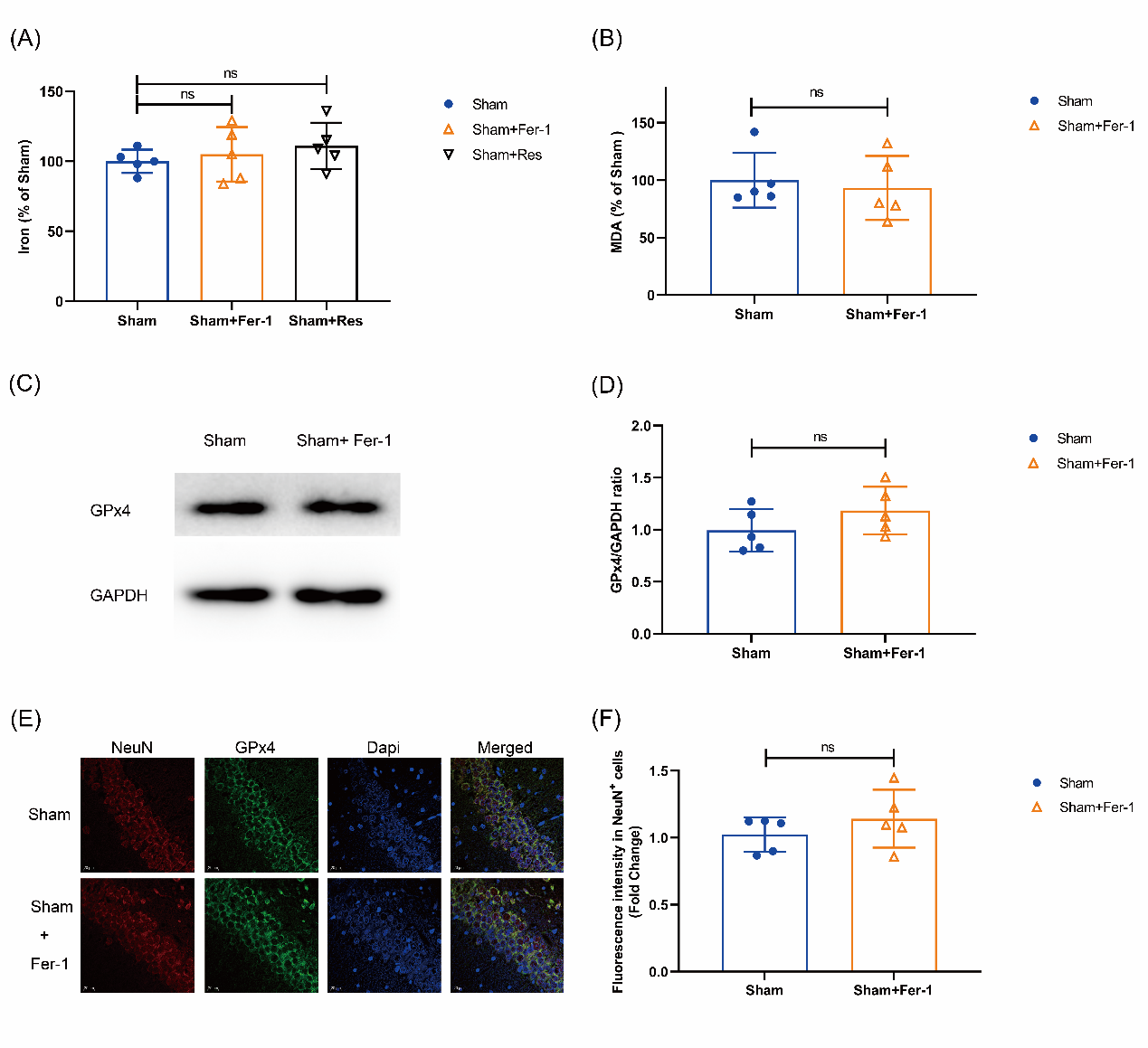
**

**Figure S1 Changes in the expression of ferroptosis markers.** (A) Statistical analysis and comparison of iron content among Sham, Sham+Fer-1, and Sham+Res groups (*n* = 5 per group). (B) Statistical analysis and comparison of MDA concentration in the Sham vs. Sham+Fer-1 groups (*n* = 5 per group). (C) Representative western blotting images (*n* = 5 per group). (D) Quantification of GPx4 levels. (E) Representative immunofluorescence staining of GPx4 (green) and NeuN (red) in the hippocampal CA1 region (*n* = 5 per group). Scale bar = 20 µm. (F) Fluorescence intensity of GPx4 level in NeuN-positive cells was quantified in the Sham and Sham+Fer-1 groups. Data represent the mean ± SD. ns: not significant. Fer-1: ferrostatin-1; Res: resveratrol; MDA: malondialdehyde; GPx4: glutathione peroxidase 4;
