# Supplementary material for: Ferroptosis contributes to hypoxic–ischemic brain injury in neonatal rats: Role of the SIRT1/Nrf2/GPx4 signaling pathway
Source: CNS Neurosci Ther. 2022 Oct 2;28(12):2268–80. doi: 10.1111/cns.13973 (PMC9627393; doi:10.1111/cns.13973)
Supplement: Supplementary file 2 — Figure S2 [file CNS-28-2268-s001.doc]

**
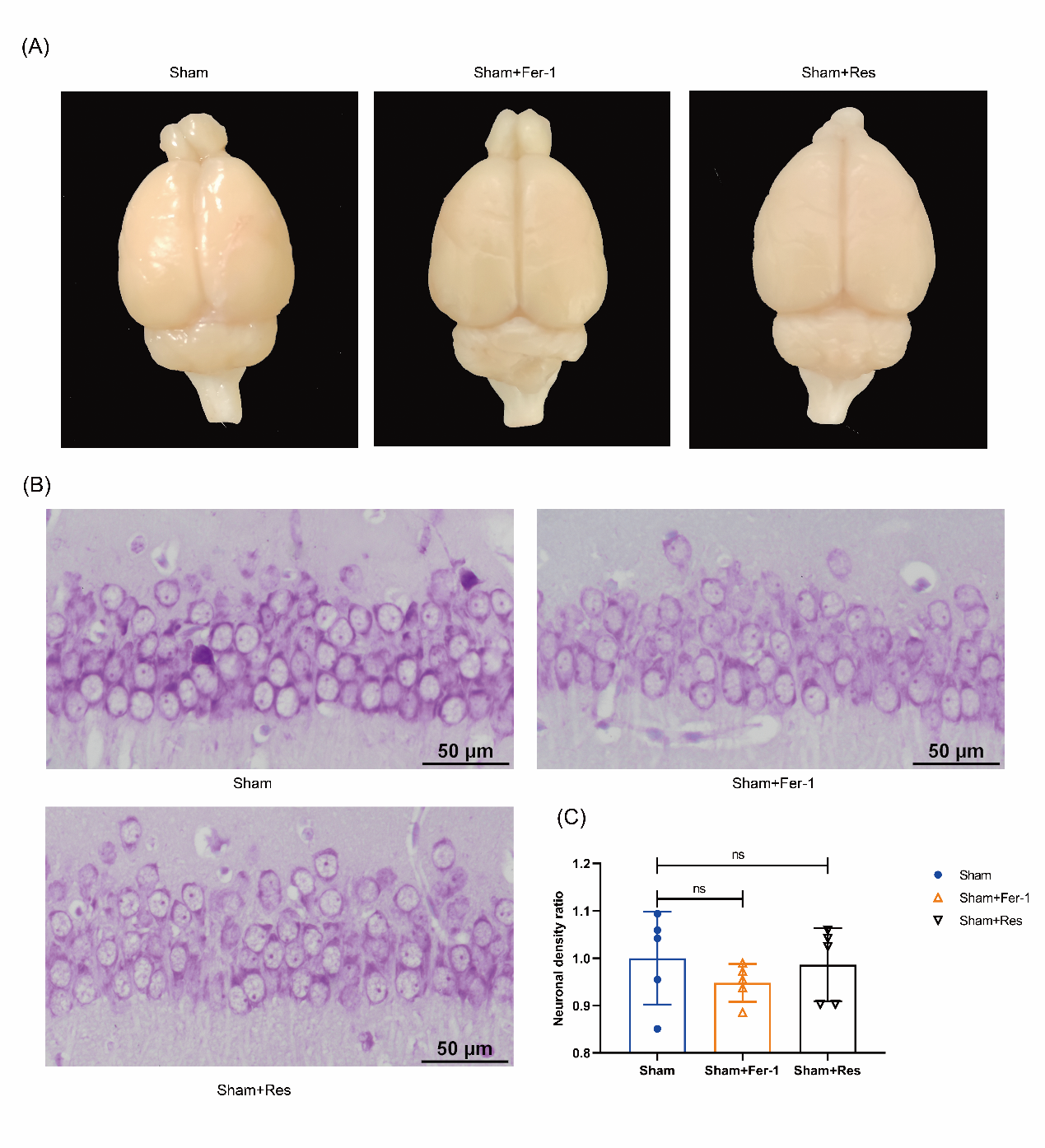
**

**Figure S2 Fer-1 or Res treatment did not significantly affect brain morphology, cell density, or hippocampal architecture of the CA1 region in Sham group rats.** (A) Representative brain morphological images of the Sham, Sham+Fer-1, and Sham+Res groups obtained on day 34 post-birth. (B) Nissl staining of the hippocampal CA1 region in the Sham, Sham+Fer-1, and Sham+Res groups on day 34 post-birth (*n* = 5 per group). Scale bar = 50 µm. (C) Neuronal density ratio in the hippocampal CA1 region (*n* = 5 per group). Data represent the mean ± SD. ns: not significant. Fer-1: ferrostatin-1; Res: resveratrol; CA1: cornu ammonis 1.
