# Supplementary material for: Ferroptosis contributes to hypoxic–ischemic brain injury in neonatal rats: Role of the SIRT1/Nrf2/GPx4 signaling pathway
Source: CNS Neurosci Ther. 2022 Oct 2;28(12):2268–80. doi: 10.1111/cns.13973 (PMC9627393; doi:10.1111/cns.13973)
Supplement: Supplementary file 3 — Figure S3 [file CNS-28-2268-s005.doc]

**
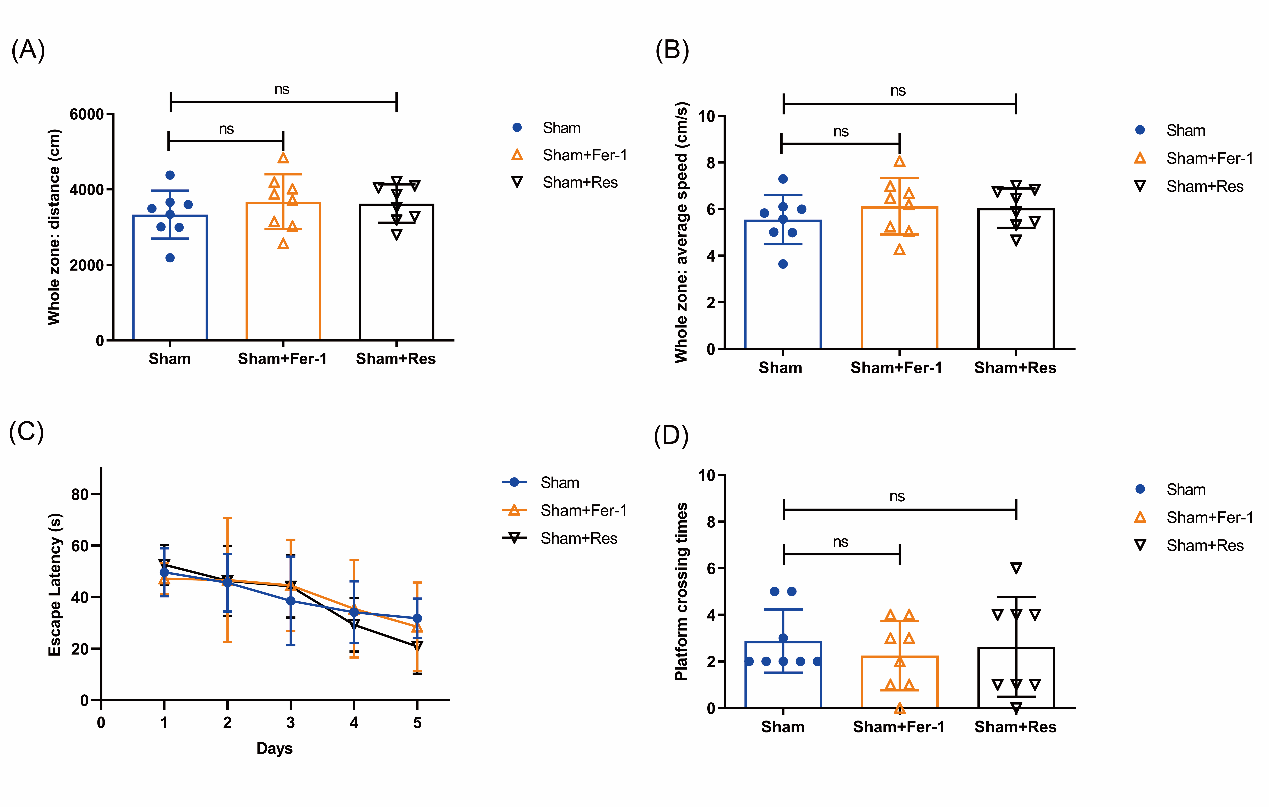
**

**Figure S3 Fer-1 or Res treatment did not significantly alter motor function or long-term learning and memory abilities** **in Sham group rats.** (A, B) Statistical results of total distance and average speed measured during the OFT indicated no significant differences in motor function among Sham, Sham+Fer-1, and Sham+Res groups (*n* = 8 per group). (C, D) Results of escape latency and platform crossing times measured during the MWM test indicated no significant differences in long-term learning or memory abilities among Sham, Sham+Fer-1, and Sham+Res groups (*n* = 8 per group). Data represent the mean ± SD. ns: not significant. Fer-1: ferrostatin-1; Res: resveratrol; OFT: open-field test; MWM: Morris water maze.
