# Supplementary material for: Ferroptosis contributes to hypoxic–ischemic brain injury in neonatal rats: Role of the SIRT1/Nrf2/GPx4 signaling pathway
Source: CNS Neurosci Ther. 2022 Oct 2;28(12):2268–80. doi: 10.1111/cns.13973 (PMC9627393; doi:10.1111/cns.13973)
Supplement: Supplementary file 4 — Figure S4 [file CNS-28-2268-s007.doc]

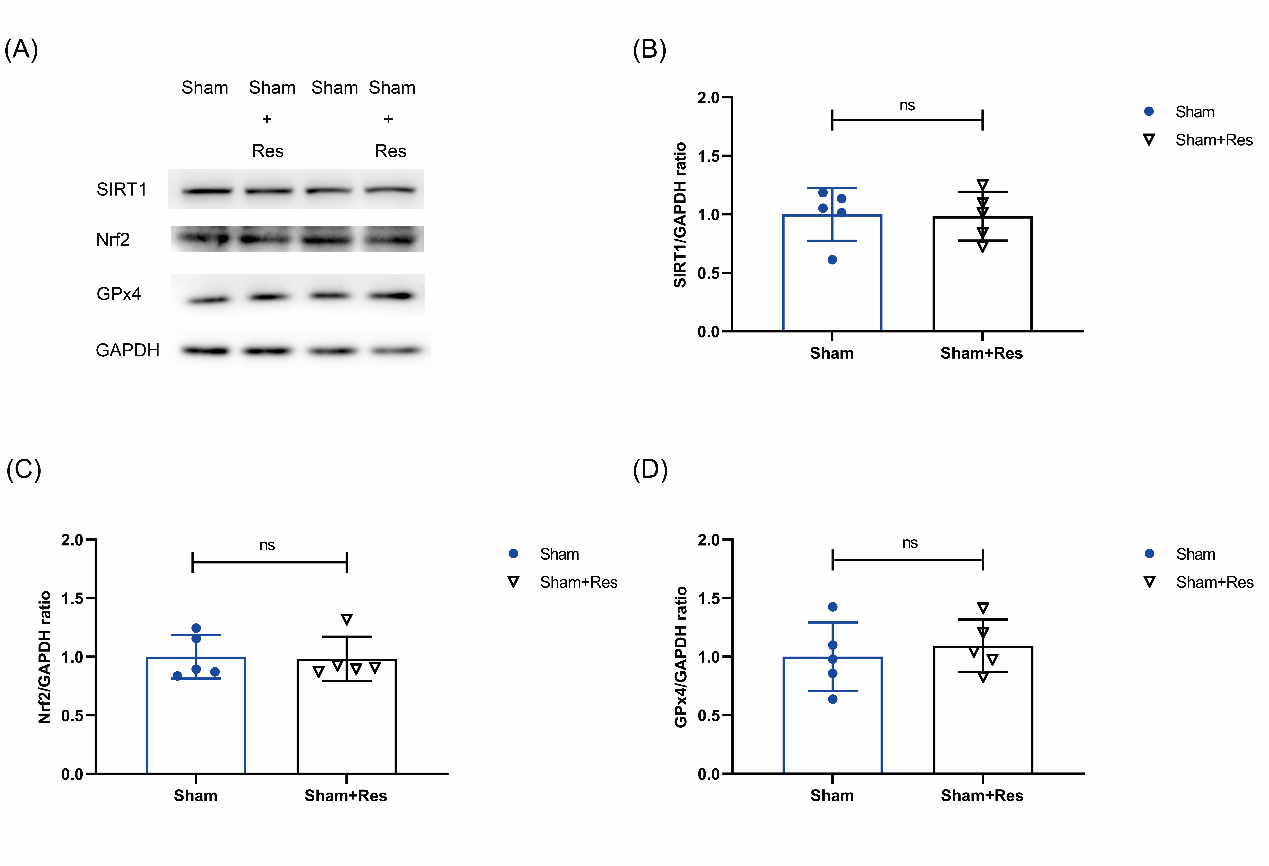


**Figure S4 SIRT1, Nrf2, and GPx4 expression in the Sham and Sham+Res groups.** (A–D) Representative (A) western blots and quantification of (B) SIRT1, (C) Nrf2, and (D) GPx4 levels. Data represent the mean ± SD. (*n* = 5 per group). Data represent the mean ± SD. ns: not significant. GPx4: glutathione peroxidase 4; Fer-1: ferrostatin-1; SIRT1: silent information regulator factor 2-related enzyme 1; Nrf2: nuclear factor erythroid-2-related factor 2; Res: resveratrol; GAPDH: glyceraldehyde 3-phosphate dehydrogenase.
