# Supplementary material for: Ferroptosis contributes to hypoxic–ischemic brain injury in neonatal rats: Role of the SIRT1/Nrf2/GPx4 signaling pathway
Source: CNS Neurosci Ther. 2022 Oct 2;28(12):2268–80. doi: 10.1111/cns.13973 (PMC9627393; doi:10.1111/cns.13973)
Supplement: Supplementary file 5 — Figure S5 [file CNS-28-2268-s006.doc]

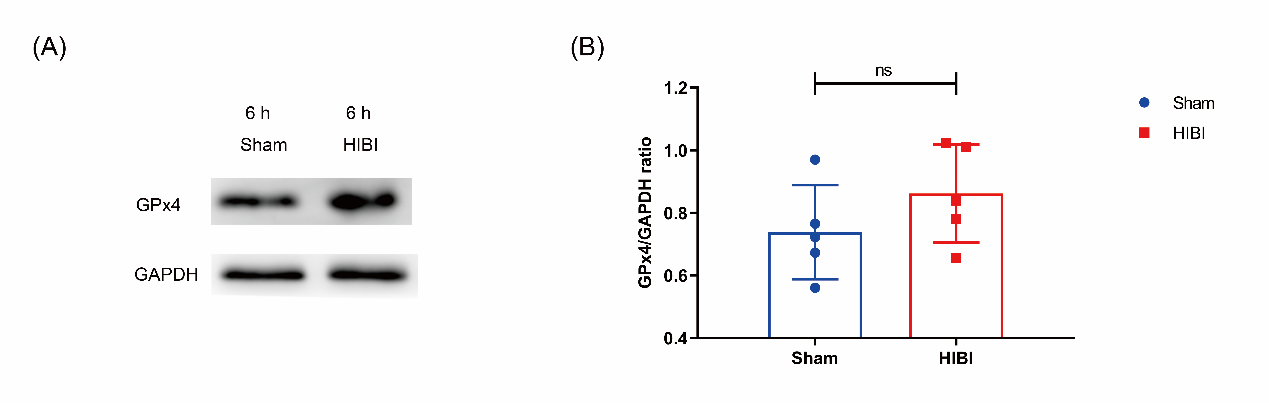


**Figure S5 Changes in in GPx4 expression at 6 h post-HIBI.** (A) Representative western blot images (*n* = 5 per group). (B) Quantitative analysis of GPx4 levels. Data represent the mean ± SD. ns: not significant. HIBI: hypoxic-ischemic brain injury; GPx4: glutathione peroxidase 4; GAPDH: glyceraldehyde 3-phosphate dehydrogenase.
