# Supplementary material for: Ferroptosis contributes to hypoxic–ischemic brain injury in neonatal rats: Role of the SIRT1/Nrf2/GPx4 signaling pathway
Source: CNS Neurosci Ther. 2022 Oct 2;28(12):2268–80. doi: 10.1111/cns.13973 (PMC9627393; doi:10.1111/cns.13973)
Supplement: Supplementary file 6 — Figure S6 [file CNS-28-2268-s004.pdf]

Full unedited gel/blot for Figure 2A

GPx4

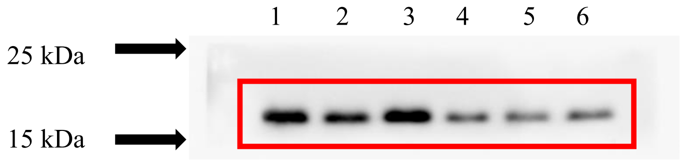

GAPDH

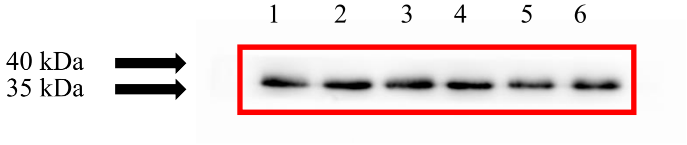

1 = 12 h Sham 2 = 12 h HIBI 3 = 24 h Sham 4 = 24 h HIBI 5 = 48 h Sham 6 = 48 h HIBI

Full unedited gel/blot for Figure 2F

GPx4

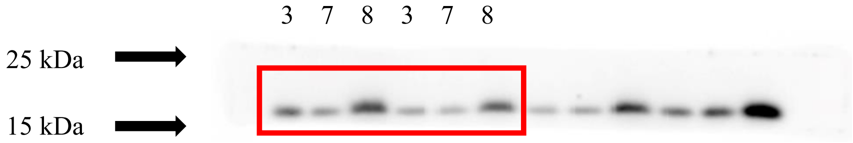

GAPDH

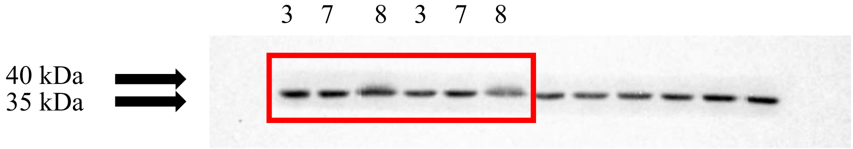

3 = Sham 7 = HIBI 8 = HIBI+Fer-1

Full unedited gel/blot for Figure 6A

SIRT1

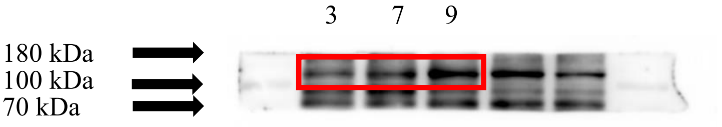

Nrf2

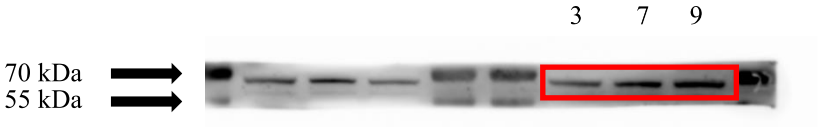

GPx4

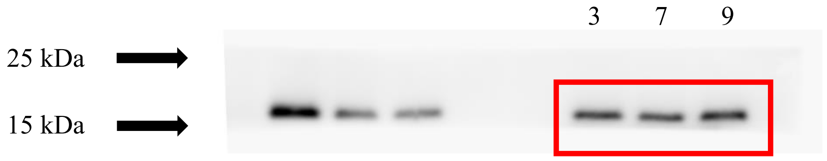

GAPDH

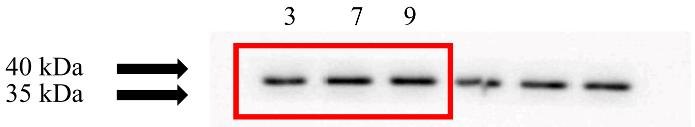

3 = Sham 7 = HIBI 9 = HIBI+Res

Full unedited gel/blot for Figure S1C

GPx4

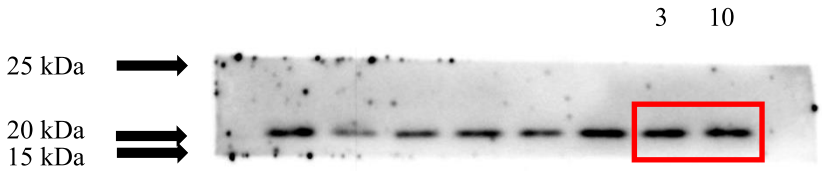

GAPDH

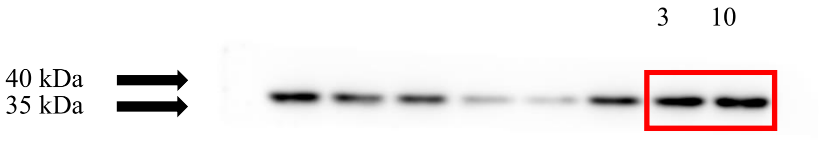

3 = Sham 10 = Sham+Fer-1

Full unedited gel/blot for Figure S4A

SIRT1

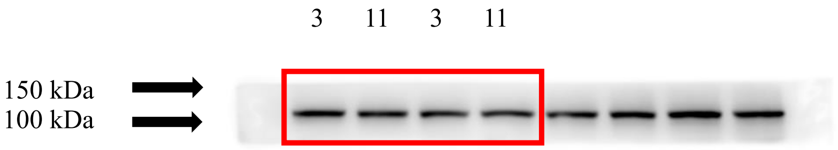

Nrf2

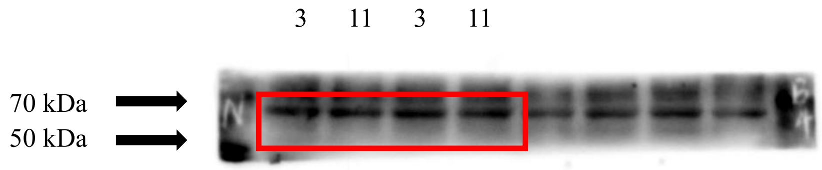

GPx4

3 11 3 11

25 kDa
20 kDa
15 kDa

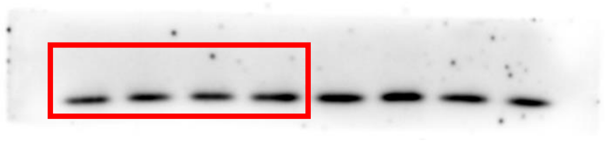

GAPDH

3 11 3 11

40 kDa
35 kDa

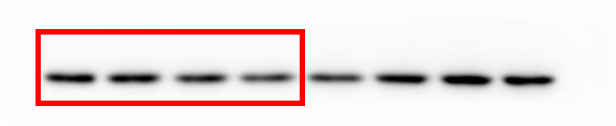

3 = Sham 11 = Sham+Res

Full unedited gel/blot for Figure S5A

GPx4

12 13

25 kDa
15 kDa

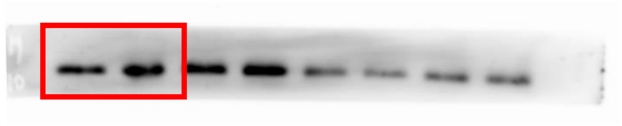

GAPDH

12 13

40 kDa
35 kDa

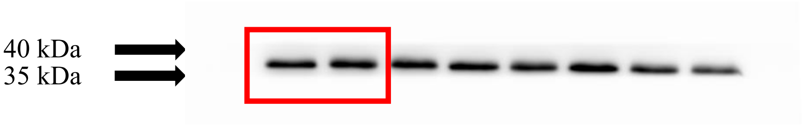

12 = 6 h Sham 13 = 6 h HIBI
