# Supplementary material for: Ferroptosis contributes to hypoxic–ischemic brain injury in neonatal rats: Role of the SIRT1/Nrf2/GPx4 signaling pathway
Source: CNS Neurosci Ther. 2022 Oct 2;28(12):2268–80. doi: 10.1111/cns.13973 (PMC9627393; doi:10.1111/cns.13973)
Supplement: Supplementary file 7 — Tables S1‐S2 [file CNS-28-2268-s003.doc]

# Ferroptosis Contributes to Hypoxic–Ischemic Brain Injury in Neonatal Rats: Role of the SIRT1/Nrf2/GPx4 Signaling Pathway

# Chang Li, Ziyi Wu, Hang Xue, Qiushi Gao, Yahan Zhang, Changming Wang, Ping Zhao

**Supplementary Information**

**Table S1 General characteristics** **(**mean ± SD**)**

|  | Fate after HIBI | | |  | Body weight (g) of the survivors | | |
| --- | --- | --- | --- | --- | --- | --- | --- |
|  | Total | Dead | Mortality% |  | 7 days old | 14 days old | 34 days old |
| Sham | 10 | 0 | 0 |  | 12.9±0.6 | 30.73±1.07 | 90.61±1.66 |
| Sham+Fer-1[[1]](#footnote-2)† | 10 | 0 | 0 |  | 12.77±0.85 | 30.96±1.56 | 89.61±2.31 |
| Sham +Res[[2]](#footnote-3)‡ | 10 | 0 | 0 |  | 12.85±0.95 | 31.6±1.88 | 89.65±1.73 |

**Table S2 Weight ratio of left/right cerebral hemispheres** **(**mean ± SD**)**

|  | Left cerebral weight (mg) | Right cerebral weight (mg) | Left/right (%) |
| --- | --- | --- | --- |
| Sham | 480.6±20.82 | 479.4±24.08 | 100.3±1.55 |
| Sham+Fer-1 | 479.4±18.22 | 483.6±14.38 | 99.22±5.33 |
| Sham + Res | 487.0±21.41 | 488.6±22.91 | 99.69±0.74 |

1. ^†^ Fer-1: ferrostatin-1 [↑](#footnote-ref-2)
2. ^‡^ Res: resveratrol [↑](#footnote-ref-3)
